# Power, class, and gender in dementia care: Stories of carer precariousness from culturally and linguistically diverse female family carers in Australia

**DOI:** 10.1177/14713012251342059

**Published:** 2025-05-11

**Authors:** Teddy Nagaddya, Ruth Brookman, Olivia Maurice, Celia B. Harris

**Affiliations:** School of Social Sciences, 6489 Western Sydney University, Australia; The MARCS Institute for Brain, Behaviour and Development, 6489 Western Sydney University, Australia

**Keywords:** dementia, female carers, power, class, gender, and precariousness

## Abstract

Dementia care provision is a global challenge. However, dynamics to provide care to a family member living with dementia in their home are far more complex. Evidence suggests that dementia care among culturally and linguistically diverse (CALD) communities is largely offered by family members within communities. But at a family level, care happens at the intersection of unequal gender relations, complex cultural constructions of dementia, and longstanding family values and traditions. While these dynamics show the intersection of power, class, and gender, these aspects have not gained widespread currency in dementia policy plans. Yet they shape the identities and social positioning of carers and consequently carer precariousness. This article reveals that the role of caring for family members living with dementia is embedded in complex power structures that stigmatise the identities of carers and those cared for, reproduces gendered social hierarchies, exacerbate economic uncertainties, and diminish the merits of filial piety - a valued cultural practice. By centering the voices of female carers from a CALD background, the authors highlight the need for policymakers to devote particular attention to how the intersection of the diverse cultural environments with dementia care at a family level induce carer insecurities and vulnerabilities - precariousness. This calls for an emancipatory dementia policy agenda that values the lived experiences of female carers’ cumulative disadvantage over the course of providing care.

## Introduction

The state of precariousness for family carers of people living with dementia is defined as the experience of vulnerabilities, uncertainties, and insecurities associated with providing care (Hillman et al., 2023). Precariousness manifests as financial fragility due to inconsistent labour participation, feelings of social isolation, limited family support, psychological distress and overall poorer quality of life – all of which are exacerbated by gender and class. Yet within culturally and linguistically diverse (CALD) communities, which tend to have a collectivistic orientation (Baker et al., 2016), informal care provision to family members with chronic conditions remain most typically the premise of women. In such cases, informal caring is viewed as less intellectually challenging or pressing and with a lower social status than the financial roles of men who are viewed as bread winners for families (DeForge et al., 2017). Yet, unlike individualistic populations, collectivistic cultures tend to foreground caring as a gendered and family obligation. In the context of familial dementia care, these gendered and class values, disadvantage female CALD carers in several ways.

Moreover, dementia is often a highly stigmatised condition within culturally and linguistically diverse minorities (Allam et al., 2023). Stigma in this context entails stereotyping and labelling those living with dementia as deviant from what is culturally considered normal and leads to status loss and discrimination (Link & Phelan, 2001). Most importantly, dementia is the leading cause of disability in old age particularly among minority groups (Avan & Hachinski, 2021; Lisko et al., 2021; Prince et al., 2016; Tuffour & Ganga, 2024). For instance, in Australia, the population of older people from minority groups living with dementia in communities is growing (Shatnawi et al., 2023). This growth has stimulated public policy conversations on how care can be organised at community level (Chelberg, 2023).

It is estimated that 37% of Australia’s ageing population are foreign born (AIHW, 2023) – indicating a rapidly diversifying profile of older people. Projections further show that by 2056 there will be a predominantly non-European older population (Wilson et al., 2022). Worth noting is that diversity drives the public sector to seek solutions to ensure equitable quality of care and enhanced care experiences for culturally and linguistically different populations.

Recent data shows that in Australia, dementia is the second leading cause of burden of disease after coronary heart disease (AIHW, 2024). It accounts for 10% of all deaths in Australia with significantly higher mortality rates observed among men than women. Of all the deaths reported between September 2016 and December 2017, a significantly higher rate of dementia (43%) was observed among men from non-English speaking background compared to those who were Australian born (38%). However, the complexities associated with dementia care within communities and among culturally and linguistically diverse populations of older people are not well articulated (Low et al., 2019).

Research shows that, in Australia, 2 in 3 older people with dementia are living at-home in the community as opposed to within residential care (AIHW, 2021). While the choice to receive care within the community reduces the risk of social isolation and promotes ageing in social groups (Odzakovic et al., 2021), it also increases the extent to which care is the responsibility of family members and may increase the likelihood of carer precariousness.

Dementia care policy plans have not paid much attention to the extent to which precariousness is experienced by carers, and the broader socio-cultural factors shaping that state of vulnerability. For instance, the Global Plan of Action on the public health response to dementia, (2017–2025), acknowledges that national dementia plans are still underperforming in addressing the needs of carers and families. Yet there is significant and growing dependence on informal carers for dementia care support (Carter et al., 2021). For example, Australia has made tremendous progress in addressing the needs of people living with dementia and their carers through its National Dementia Action Plan 2024-2034 (Department of Health and Aged Care, 2024), that highlights eight priority actions. The plan clearly highlights the role of gender in dementia care and the negative impact of the caring role on women. However, while the plan centres the support of carers (Action 6), there is no distinction made about the complexities of informal versus formal carers and facility-based versus home based carers, nor any consideration of the family and cultural dynamics associated with care giving. This masks the needs, for instance of female CALD informal carers who are confronted with cultural and familial complexities associated with dementia care giving.

In this article, the authors seek to contribute to the understanding of carer precariousness associated with looking after a family member living with dementia. The authors point out that the role of caring for family members living with dementia is a labour of love and maintaining ongoing relationships (Todorova et al., 2016) but is also embedded in complex power structures that can stigmatise the identities of carers and those cared for, reproduce gendered social hierarchies, exacerbate economic uncertainties, and diminish the merits of filial piety. In the current research, we aimed to examine female carers' reflections on their caring role, and we analysed interviews through the lens of precariousness to examine how intersecting aspects of their identities including gender and culture may impact on their wellbeing and their relationship with their loved ones.

## Precarity in dementia care among culturally and linguistically diverse communities

Australia is increasingly becoming home to an ethnically diverse population of older people (Pham et al., 2021). However, concerns about dementia care and support services have become more pronounced, as dementia risk increases with age (Ahmad et al., 2022), and there is a need to ensure that aged care services can be tailored and suitable for this increasingly diverse older population. Ageing in the context of migration presents diverse and complex health care related challenges not only to the host country but also to migrants who must adapt to and navigate new cultural care systems (Hall, 2023; Javanparast et al., 2020; Khatri & Assefa, 2022). Interacting with unfamiliar care systems when managing complex conditions such as dementia exposes ethnically diverse groups of carers and those with dementia to a wide range of risks and uncertainties, which can be understood as care precarity.

Although the term precarity has predominantly been used in the literature to refer to risks and insecurities associated with labour conditions (Bourdieu, 1998), most recently it has found its way into the ageing and aged care discourse to depict the social, cultural, economic and political inequalities in late life (Fine, 2020; Gilleard & Higgs, 2020; Grenier et al., 2020a, 2020b). However, in the context of dementia care among CALD communities, precarity has been associated with the intersecting challenges associated with old age, migration and care (Hall, 2023), exclusion and failure to meet the care needs of carers (Hillman et al., 2023) and devaluing of older people living with dementia (Grenier. & Phillipson, 2023). Clearly, the discursive context of precarity in dementia care reveals the interplay of the aspects of power and social class which have consequences on the identities of those living with dementia and their carers and the emerging social relations. In this cross-sectional exploratory study, we used this concept as a lens with which to understand the reported experiences of family care partners of diverse people with dementia in an Australian context.

### Dementia care and power relations

Recognising a hierarchical structure in dementia care at family and healthcare facility level is a fundamental step to understanding carer precariousness. For instance, the shift to western-centric values of dementia care is a source of precarity to those from CALD backgrounds (Shanely et al., 2012). Partly, the insecurities and vulnerabilities experienced by carers and those in their care, is attributed to the differences in linguistic expression and cultural values to those of the dominant group within which care is provided. Undeniably, the outcome of these differences is the creation of social classes of minority groups (Sakai & Carpenter, 2011).

Recent research shows that carers of family members with dementia find the process of accessing formal care services entrenched in structural barriers such as lack of culturally sensitive services, language barrier, racial discrimination, fragmented services, lack of dementia knowledge, among others (Gilbert et al., 2022, Baghirathan et al., 2020; Berstein Sideman et al., 2022; Nielsen et al., 2021). Such barriers create contexts within which the exercise of power and control shape complex relationships and practices of caregiving. Feelings of mistrust and powerlessness within CALD communities needing dementia care become more pronounced when interacting with such hierarchical systems of care (Calsson & Pijpers, 2021). However, focusing on the barriers to accessing formal dementia care services masks our understanding of the concept of care within collectivist cultures – where care is viewed as a social obligation and has implications on the overall well-being of families and communities. The barrier lens diminishes efforts of sustainable dementia care within communities by discounting carers for family members living with dementia as ‘informal'. More problematic is that there is limited attention paid to the impact of the cross-cultural contexts within which dementia care happens. In the current research, we aimed to gain an understanding of how carers adapted to a new culturally different dementia care environment.

### Social relations, ethnicity and class in dementia care

Research on dementia care has shown how social relations, ethnicity and class interact to shape experiences of accessing dementia diagnosis, treatment and care (Bodryzlova et al., 2023; Lin et al., 2021). Jones (2017) observes that those living with dementia among minority groups are likely to avoid dementia diagnosis due to the distress and stigma associated with it. But there is need to be aware that social stigma associated with dementia is embedded in traditional class distinctions (Siette et al., 2023). For instance, historically, receiving of mental health treatment or therapies has been linked to one being from a low socio-economic background, lack of education and limited knowledge of services (Javed et al., 2021). Such class distinctions in dementia care do not only impact the experiences of accessing care but shift the burden of care from formal care providers to family members who are predominantly women or adult daughters (Sagbakken et al., 2018). Without supportive networks at both formal and informal level of care, the gendered and hierarchical nature of care provision positions women as the primary carers. But with the complexity and demanding nature of dementia care (WHO, 2021), those providing support to family members often experience physical, social and mental health challenges. In the current research, we focused on articulating how social relations were impacted upon by providing dementia care

## The current study

We aimed to understand the experiences of female informal caregivers from a CALD background, providing care to family members living with dementia. Using a thematic analysis approach, we studied the social relations and multi-faceted nature of vulnerabilities and insecurities (precariousness) that were associated with the caring role. By identifying the challenges that caregivers experienced in navigating their personal responsibilities such as paid work and informal caregiving obligations, the study findings will contribute to a body of literature that seeks to provide a holistic approach to enhancing informal carers’ well-being.

## Method

This qualitative study was approved by the Western Sydney University human research ethics committee [approval: 14858]. Informed consent was obtained from all participants prior to their involvement in the study, in accordance with ethical guidelines.

## Study design and context

This present qualitative study took a human-centred approach and emphasised the importance of lived experience and was conducted under a social constructionism paradigm that is principally concerned with the social, cultural and economic processes by which informal caregivers come to describe and explain their realities of providing dementia care (Berger & Luckmann, 2016, pp. 110-122). The study employed a cross-sectional exploratory design using semi-structured interviews with family carers of people living with dementia.

The study was conducted in Sydney, Australia which is a multicultural city, where carers with a CALD background have lower levels of dementia literacy, and experience later diagnosis than non-CALD Australians (Australian Government NHMRC National Institute, 2020). Most family care partners in Australia provide care for 60 or more hours per week (AIHW, 2021). As the prevalence of dementia is increasing in Australia, so also is the demand for family care partners, resulting in recent policy efforts to increase the support available to carers (Engel et al., 2022).

## Participants

This cross-sectional study included data from the final interview with care partners from the longitudinal cohort from Brookman et al. (2025). The full cohort of participants were 13 female care partners (*Mean age* = 62.2 years) and 2 male care partners (73.5) for 17 people with a dementia diagnosis. Care partners were an English-speaking purposive sample approached and recruited by email and phone via networks, including the Step Up for Dementia database (Jeon et al., 2021). However, the research reported here focused on only 5 female care partners who identified as CALD due to their unique experiences which are under-explored. So, from a social justice perspective, it is vitally important to give these care partners a voice, whose lived experiences will inform a larger-scale study. Care partners had diverse caring responsibilities and arrangements. One person with dementia was in residential care at the time of the interview; and another was receiving palliative care at home. Two care partners cared for more than one person with dementia e.g., two parents. Most care partners cared for their partner and the remainder cared for parent/s. Diagnoses of dementia had occurred between 2016 and 2022, most commonly with Alzheimer’s disease (Table 1). Most care partners lived full-time with the person with dementia, one care partner spent most of the year overseas caring remotely and spent approximately one month onsite; two care partners lived half the week at their own home and half with their parent with dementia. No participants withdrew from the study.

**Table 1.** Dementia diagnosis at the time of recruitment.

Dementia type	Count (*n*)
Alzheimer’s disease	6
Dementia (general/unknown)	2*
Vascular dementia	3*
Cortico-basal degeneration	1
Frontotemporal dementia	1
Early onset dementia	2
Vascular dementia and Alzheimer’s disease Not provided	1 1
Total	17

*Another person with dementia was initially diagnosed with this type of dementia but was subsequently re-diagnosed as having Alzheimer’s disease.

*Note.* One person with dementia had a dual diagnosis of vascular dementia and Alzheimer’s disease. Other PLWDs had diagnosed comorbidities such as autism spectrum disorder and Parkinson’s disease.

## Procedure

### Care partner interviews

Online interviews (60–90 minute) were conducted between a member of the research team (female) and care partners between January and March 2023, via Zoom teleconference. All questions were open-ended and phrased conversationally. Example questions included: ‘Would you say your cultural background favours the individual or the collective?’ and ‘Has your cultural upbringing influenced your decision to take on carer responsibilities?’. Participants received an AUD $50 gift card following the interview.

The interviews were conducted by the second female author (PhD/MClinPsy), employed as a research fellow and registered psychologist with clinical training and extensive field work experience interviewing older adults and caregivers of people with dementia.

### Interview data analysis approach

The interviews were audio and video recorded and transcribed using Otter.ai software with manual checking and removal of identifying information. No software platforms were used to manage the data. The interview transcripts were analysed using a thematic analysis approach (Clarke & Braun, 2017) that involved five steps: (1) becoming familiar with the data; (2) generating initial codes; (3) identifying themes; (4) reviewing and refining themes; (5) defining and naming final themes and (6) producing the report. For steps 1 and 2, transcripts were coded by the first author (female) (PhD), who was employed as a Lecturer in social science and a trained gerontologist. Before migrating to Australia, she had exposure to both Western and non-Western-centric values of elderly care. This lived experience was important in maintaining a neutral position during data analysis and an empathetic lens to participants’ lived experiences. While themes were not identified in advance but were derived from the data, the concept of precarity was used as a lens with which to understand the reported experiences of family care partners with a CALD background. Following initial coding of all transcripts, the first female author grouped codes into theme clusters, reviewed themes, and finalised them (steps 3–6). Participants did not provide feedback on the data (see Supplemental Materials, S1, for a completed COREQ 32 [Tong et al., 2007] checklist).

## Findings and discussion

**Figure 1.**
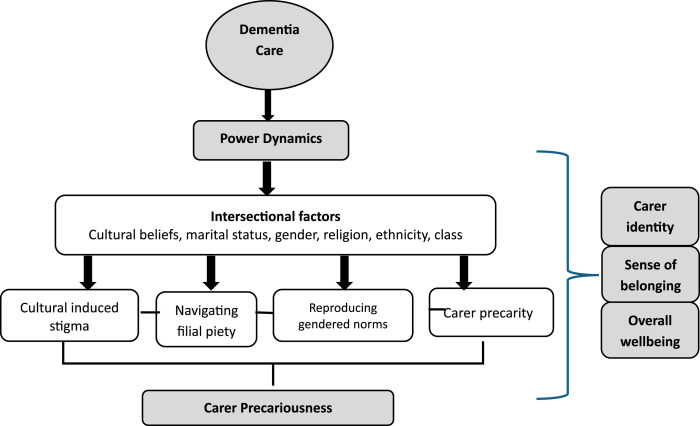
Precariousness in dementia care among female carers from a culturally and linguistically diverse background.

Carer precariousness is an underexplored aspect in dementia care research, yet it has a debilitating impact on carers’ identity, sense of belonging and overall well-being. As summarised in Figure 1 below, four themes from the study emerged to demonstrate the precarity trap that position caregivers from CALD backgrounds in a state of cumulative disadvantage resulting in carer precariousness throughout the life course of care provision. The themes include ‘culturally induced stigma’, ‘navigating filial piety’ ‘reproduction of gendered norms in dementia care’ and carer’s precarity, which manifested as carer precariousness. Findings showed that carers’ struggles are embedded in power structures that are shaped by the intersection of gender, socio-cultural and material conditions.

### Cultural induced stigma of carer and person living with dementia

Participants in this study predominantly spoke of carer precarity through the cultural constructions of dementia that stigmatised those living with the condition and their carers and discredited their identities. Culturally, dementia, as revealed by some participants, was viewed as a condition where one ‘loses their mind’ and referred to as ‘crazy man’ – which was a mark of disgrace that exacerbated social distance. The condition was not only associated with shame, loss of dignity and powerlessness but it also led to the invisibility of those living with dementia as one participant from a Greek background revealed:

“I feared stigma, I feared embarrassment, I feared judgement. And so, I refused to disclose my husband’s state of health. It's something to be ashamed of. Soon as he [her dad] was diagnosed with early mild dementia, every man and his dog wanted to come and see what it was like to see a man lose his mind” (P01).

It’s not only carers that felt a sense of alienation, but they also reported that their family members living with dementia experienced a devalued social identity and were at a risk of public ridicule because they were viewed as behaviourally different from the norm.

Similarly, notions of a dehumanising community discourse towards people living with dementia prevailed. This left some carers with stigmatised identities. One emotionally drained participant recalled the way her dad was viewed by the community and the sense of powerlessness she felt at that time:

“So, then it's like, all of a sudden, the community started wanting to visit him [Dad], because it became like he was ......an animal on display. As a carer, I felt exhausted, lost and alienated because not only was I carrying community stigma, cultural expectation, the role of the advocate and the carer, I did not feel that I could ask anyone for help”. (P01)

Based on the carers’ narratives, community and cultural constructions of dementia and the labels associated with the condition, lead to stigmatised identities and an assumed loss of status.

### Negotiating reciprocal filial piety in dementia care

Some participants spoke of the on-going culture of filial piety. Culturally, unlike sons, elder daughters were expected to care for their parents as a sign of respect as one participant put it ‘...you should honor your parents’ (P06) and another participant was quick to highlight how she had been socialised into this caring role:

‘I was always brought up in a way that you must look after your family. So, my first responsibility is my husband. And it's the parents and things like that. So that's why I do this regularly.’ (P06)

Another participant felt that her sense of self-determination was taken away by the dictates of social-cultural constructions of family care, ‘I didn't get asked if I wanted to be a carer… it just became an obligation’ (P01). Alluding to similar feelings of lack of choice as a career, one participant from an Italian background reflected on her experience:

“I'm their daughter so I should just deal with whatever I need to deal with. Because that's just the way it is, you know, that I that I shouldn't have my own life or my own career or whatever” (P09)

The cultural expectations for one to care for a family member living with dementia became more evident if one was the eldest daughter in the family as revealed by one participant:

‘I'm the eldest child as well and a girl. So, there is an expectation that the children look after the parents, and particularly me, because I'm the eldest and female’ (P09)

Being an elder daughter in the family and failing to provide care led to feelings of guilt and labels of not being a good daughter or child. While this experience was not common among the majority of participants, one carer from an Indian background shared her caring experience and the motivation to taking on the role.

‘I'm not only driven by guilt, I think it's just, it's more empathy or sympathy,” It's sometimes verbalised like, a good daughter would do this, a good daughter would do that, a good child would do this’ (P15).

Guilt was expressed in different ways. One participant who was looking after her mother from Poland was asked

***Interviewer:*** I'm wondering if her cultural heritage impacts your caring role?

***Carer:*** Guilt… The guilt of when we were living there, and if I organised a support worker to come in, just so we could simply go out to our local little restaurant and have a meal or something just to have a break. When I was there full time, my mother would get very upset and you know, just really put the guilt trip on (P13)

The collective and relational nature of dementia care through the notion of filial piety – which is a valued cultural practice, left female carers feeling disempowered.

### Reproduction of gendered norms in dementia care

Based on some participants’ narratives, dementia care was a precarity trap as it reproduced gendered social hierarchies that reinforced women’s subordinate position. Female carers were cognisant of the impact of the intersection of gender with culture and religion in shaping and preparing them for the caring role. But this situation left some of them feeling disempowered as one participant revealed.

“…as a Middle Eastern woman, I've been conditioned culturally to serve the family and including the men. So, when my husband was diagnosed, I jumped straight in, I acted out of duty and obligation, but I struggled to engage and cope with the imposed role” (P01).

Despite embracing their informal caring roles, one carer recognised the consequences of unequal power relations at family level in navigating dementia care. One participant recounted; “I wasn't given a choice about caring and family didn't step up to support me.” *(P01)*. In addition, caring within a new country context induced mixed feelings about carer vulnerability, identity, patient health outcomes, and quality of life.

“As a vulnerable Muslim woman [from the Middle East] living in the West. I have struggled to come to terms with the caregiving role. Does the person with dementia experience quality care when the partner is caring out of obligation? The care giving role has left me feeling highly strung, fearful, sensitive, vulnerable not to mention socially isolated, disempowered and extremely protective of my husband.” (PO1)

Caring for a family member living with dementia in a different cultural context, to some extent showed a conflict of caring cultures and the continuous reproduction of an underclass of female carers.

### Carers’ precarity induced by cultural expectations

Recognising that dementia care was such a demanding role and time consuming, some participants viewed the caring role as sacrificing their life goals and making them insecure. Feeling frustrated and a sense of powerlessness, a participant summed this experience of precariousness as “*the woman is always the martyr” (P09*). The influence of patriarchal and cultural values ignited a sense of invisibility and relationships of control that did not value women’s economic participation and hence leaving female carers in precarious conditions. Carers’ diminishing economic rights and citizenship heightened their precarity. A participant looking after her husband with dementia narrated her experience of precariousness as:

“I didn't sign up for this. And I felt I was losing myself, my sense of self and support and purpose. I was forced to leave my job that I love. And the caring role consumed me. And I felt disillusioned, and I was angry.” (P01)

The caring role led to a huge financial burden and locked women in a cycle of gendered poverty. Yet, failure to provide care induced feelings of guilt. One participant looking after her mother with dementia and elderly dad highlighted that:

“So, I've had six months, more than six months off work now. I actually just went back to work this week, just one day a week. And yeah, and that was an enormous financial burden. So, I would have to give up my entire life to look after one, let alone two of them. It would mean giving up my family and career and everything else.” (P09).

Caring for a family member living with dementia was a full-time responsibility that hindered female carers from achieving their personal and professional aspirations. Care was embedded in individualism, material and cultural values that threatened personhood.

## Discussion and conclusion

As demonstrated in this article, providing dementia care to family members at a family level is such a challenging experience. While this experience is not unique to female carers from a CALD background, we argue that care in CALD communities happens at the intersection of unequal gender relations, complex cultural constructions of dementia, and longstanding family values and traditions that expose carers to precarious conditions that manifested as stigma, filial values, gendered norms and precarity. Conditions of precariousness were reported to be amplified by gender intersecting with migration, social class, position in the family and marital status. But the consequences influenced carer identity, sense of belonging and overall well-being of carers.

Across several participants and consistent with the literature on mental health, providing dementia care is associated with stigma and loss of status (Rewerska-Juśko & Rejdak, 2020; Siette et al., 2023). Carers become victims of secondary stigma which emanates from caring for those considered to be societal deviants. These culturally given social categories associated with dementia reproduce social classes and systems of power that shape how care is provided. With a devalued carer social identity, access to support systems, social, economic and political power in decision making processes is hindered due to social stratification. Carers become constantly trapped in a re-stigmatisation cycle and consequently carer insecurities and vulnerabilities. Yet efforts to support carers tend to focus on individual lived experiences of stigma (Gilbert et al., 2022) without paying attention to the relationships and broader cultural structures within which this devaluation occurs. The full weight of this understanding is often viewed as inconsequential because the human differences against which those living with dementia and their carers are stigmatised, are taken for granted and become socially acceptable norms within some CALD communities. But when carers feel ‘embarrassed and judged’ because of caring for a ‘crazy’ family member (societal labels and stereotypes) – as participants expressed, then any dementia policy actions to support carers should interrogate the social processes within which these beliefs and labels are constructed and their impact on carer precariousness.

When such social processes are critically examined, then dementia care planners will find it easy to identify where the responsibility to fight stigma lies. This is vitally important if indeed sustainable support for carers is to be achieved. As observed by Link and Phelan (2001), stigma occurs in spaces where power is exercised. When carers develop beliefs of loss of status, it reveals existing power relations based on socially acceptable human differences associated with dementia as a condition and dementia care. However, the sources of this stigma and the structural fabric within which it is constructed remain less explored in the dementia care discourse. Moreover, stigmatisation influences how carers access social support, economic opportunities and decision-making power.

The findings further illustrated how the culture of filial piety, which is a moral and valued cultural practice (Wang et al., 2023), motivated the provision of dementia care but also increased the risk of carer precariousness. In the context of dementia care among CALD communities, filial values increased the burden of caregiving and mental health stress among carers regardless of the relationship they had with the person living with dementia (Dad or husband). Female carers felt trapped in the caring role rather than choosing to provide dementia care. They had to sacrifice their socio-economic lives for family members living with dementia. Consistent with international literature, Hall (2023) refers to this situation as carer precarity. Predominantly, filial values negatively impacted women in terms of reinforcing their subordinate position and power disparities at family level. Unfortunately, this level of marginalisation over the course of providing care remains silent in policy actions aimed at supporting carers. Yet, long-term dementia care provision has the potential of creating an underclass of female carers, hence heightening feminised poverty.

Similarly, carer precariousness was attributed to the gendered nature of caring work (Akkan, 2020), that left carers with no sense of agency. However, the situation became complex where one had to look after their spouse and their dad in their capacity as adult daughters. The combination of care identities and responsibilities positioned carers in a vulnerable and insecure position. Carers’ lives revolved around meeting the care needs of family members but with no one to meet the needs of carers. While this is not a new finding, the experience of providing dementia care was characterised by carer invisibility due to experiences of social stigma and the intersection of gender with social class, religion and marital status.

On the other hand, female carers experienced oppression and marginalisation through cultural systems of care that reduced carers participation in the labour market - precarity. Dementia care work in the context of the studied CALD families counted for nothing as female carers experienced cumulative disadvantage associated with socio-economic exclusion. The demanding nature of dementia care leaves no flexibility for carers to engage in meaningful paid labour. Yet there are no social benefits linked to dementia informal care work. This is well-aligned with feminist research that has clearly articulated the inequalities associated with the caring role (Nadasen, 2021). Characterised as unpaid labour, caring roles as argued by feminists are centrally organised under structural systems of unequal social relations. The situation is aggravated in the context of CALD communities where care follows the justice principles of being a social good and social process (Akkan, 2020).

## Conclusion, limitations and future research direction

In conclusion, the relationship between dementia care provision and carer precariousness needs to be given pivotal consideration in the dementia care support policy conversations that focus on culturally and linguistically diverse communities. While the dimensions of precariousness discussed in this article do not only apply to CALD communities neither assume homogeneity of communities, the significance of cultural systems of power in dementia care cannot be underestimated. However, achieving carer socio-economic security and overall well-being will require a co-design approach to dementia care solutions but also finding ways in which female carers can reconcile their paid work commitments and care obligations. This will allow the integration of family members’ views and carers’ lived experiences of disadvantage over the period of care provision. There is need to revisit the dementia welfare carer regimes at family level by shifting the focus from addressing psychosocial needs only to articulating how the intersecting stratification cultural processes that shape care provision, can alter socio-economic inequalities among carers. This may require research that adopts a phenomenological study to interrogate the interaction of power and class with gender among carers of people living with chronic conditions in later life.

One of the limitations of this study is that the findings cannot be generalised to the wider community of female carers from a CALD background. However, the lived experiences presented in this article provide relevant baseline information for a larger-scale study. Therefore, to have a broader understanding of carer precarity in the context of dementia, further research should be conducted on a larger and gender diverse sample of dementia carers from a CALD background.

## Supplemental Material

Supplemental Material - Power, class, and gender in dementia care: Stories of carer precariousness from culturally and linguistically diverse female family carers in Australia Supplemental Material for Power, class, and gender in dementia care: Stories of carer precariousness from culturally and linguistically diverse female family carers in Australia by Teddy Nagaddya, Ruth Brookman, Olivia Maurice and Celia B. Harris in Dementia

## References

[bibr1-14713012251342059] Ahmad M. van den Broeke J. Saharso S. Tonkens E. (2022). Dementia care-sharing and migration: An intersectional exploration of family carers’ experiences. Journal of Aging Studies, 60, Article, 100996. DOI:10.1016/j.jaging.2021.100996 35248317

[bibr2-14713012251342059] Akkan B. (2020). An egalitarian politics of care: Young female carers and the intersectional inequalities of gender, class and age. Feminist Theory, 21(1), 47–64. DOI: 10.1177/1464700119850025.

[bibr3-14713012251342059] Allam I. Gresham M. Phillipson L. Brodaty H. Low L. F. (2023). Beliefs around help-seeking and support for dementia in the Australian Arabic speaking community. Dementia, 22(5), 995–1009. DOI: 10.1177/14713012231166170. 36990452 PMC10262330

[bibr5-14713012251342059] Australia Institute of Health and Welfare (AIHW) (2023). Older Australians: Culturally and linguistically diverse older people . Available at: https://www.aihw.gov.au/reports/older-people/older-australians/contents/population-groups-of-interest/culturally-linguistically-diverse-people

[bibr7-14713012251342059] Australian Institute of Health and Welfare (AIHW) . (2021). Dementia in Australia 2021: Summary report. Australian Government. Available at:. https://www.aihw.gov.au/reports/dementia/dementia-in-aus/contents/about.

[bibr16-14713012251342059] Australian Institute of Health and Welfare (AIHW) (2024). Dementia in Australia. (2024). Australian Government. Available at: https://www.aihw.gov.au/reports/dementia/dementia-in-aus/contents/summary

[bibr8-14713012251342059] Avan A. Hachinski V. (2021). Stroke and dementia, leading causes of neurological disability and death, potential for prevention. Alzheimer's and Dementia: The Journal of the Alzheimer's Association, 17(6), 1072–1076. DOI: 10.1002/alz.12340. 34057294

[bibr62-14713012251342059] Baghirathan S. Cheston R. Hui R. Chacon A. Shears P Currie K. (2020). A grounded theory analysis of the experiences of carers for people living with dementia from three BAME communities: Balancing the need for support against fears of being diminished. Dementia, 19(5), 1672–1691. DOI:10.1177/1471301218804714 30318901

[bibr9-14713012251342059] Baker A. E. Procter N. G. Ferguson M. S. (2016). Engaging with culturally and linguistically diverse communities to reduce the impact of depression and anxiety: A narrative review. Health and Social Care in the Community, 24(4), 386–398. DOI: 10.1111/hsc.12241. 25939369

[bibr10-14713012251342059] Berger P. Luckmann T. (2016). The social construction of reality. In Social theory re-wired (pp. 110–122). Routledge.

[bibr56-14713012251342059] Berstein Sideman A. Al-Rousan T. Tsoy E. Pina Escudero S. D Pintado-Caipa M. Kanjanapong S , et al. (2022). Facilitators and barriers to dementia assessment and diagnosis: Perspectives from dementia experts within a global health context. Frontiers in neurology, 13. DOI:10.3389/fneur.2022.769360 PMC8997042 35418934

[bibr11-14713012251342059] Bodryzlova Y. Kim A. Michaud X. Andre C. Belanger E. Moullec G. (2023). Social class and the risk of dementia: A systematic review and meta-analysis of the prospective longitudinal studies. Scandinavian Journal of Public Health, 51(8), 1122–1135. DOI: 10.1177/14034948221110019. 35815546 PMC10642219

[bibr61-14713012251342059] Bourdieu Pierre (1998). Acts of resistance (p. 30). New York: New Press.

[bibr59-14713012251342059] Brookman R. Lipson-Smith R. Maurice O. Mcllwain N. Hofstaetter L. DiGiacomo M Harris C.B. (2025). Caring for people with dementia: Mapping the experience and journey from diagnosis. The Gerontologist, gnaf053, 65(5). DOI:10.1093/geront/gnaf053 PMC11979749 39945461

[bibr58-14713012251342059] Calsson H. Pijpers R. (2021). Diversity-mainstreaming in times of ageing and migration: implementation paradoxes in municipal aged care provision. Journal of Ethnic and Migration Studies, 47(11). DOI:10.1080/1369183X.2020.1857231

[bibr13-14713012251342059] Carter L. O’Neill S. Keogh F. Pierce M. O’Shea E. (2021). Intensive home care supports, informal care and private provision for people with dementia in Ireland. Dementia, 20(1), 47–65. DOI: 10.1177/1471301219863580. 31349753

[bibr14-14713012251342059] Chelberg K. (2023). ‘Vulnerable Monsters’: Constructions of dementia in the Australian royal commission into aged care. International Journal for the Semiotics of Law-Revue internationale de Sémiotique juridique, 36(4), 1557–1580. DOI: 10.1007/s11196-023-09979-w. PMC10011757 37362076

[bibr60-14713012251342059] Clarke V. Braun V. (2017). Thematic analysis. The Journal of Positive Psychology, 12(3), 297–298. DOI:10.1080/17439760.2016.1262613

[bibr15-14713012251342059] DeForge R. Ward-Griffin C. St-Amant O. Hall J. McWilliam C. Forbes D. Kloseck M. Oudshoorn A. (2017). Evaluating dementia home care practices: The reification of care norms. Journal of Aging Studies, 43, 23–31. DOI:10.1016/j.jaging.2017.09.002 29173511

[bibr17-14713012251342059] Department of Health and Aged Care (2024). *National dementia plan 2024-2034*: Australian Government. Accessible via: https://www.health.gov.au/sites/default/files/2025-02/national-dementia-action-plan-2024-2034.pdf

[bibr19-14713012251342059] Engel L. Loxton A. Bucholc J. Muldowney A. Mihalopoulos C. McCaffrey N. (2022). Providing informal care to a person living with dementia: The experiences of informal carers in Australia. Archives of Gerontology and Geriatrics, 102, Article 104742. DOI: 10.1016/j.archger.2022.104742 35671552

[bibr20-14713012251342059] Fine M. (2020). Reconstructing dependency: Precarity, precariousness and care in old age. In Precarity and ageing (pp. 169–190). Policy Press. DOI: 10.51952/9781447340874.ch008.

[bibr21-14713012251342059] Gilbert A. S. Antoniades J. Croy S. Thodis A. Adams J. Goeman D. Browning C. Kent M. Ellis K. Brijnath B. (2022). The experience of structural burden for culturally and linguistically diverse family carers of people living with dementia in Australia. Health and Social Care in the Community, 30(6), e4492–e4503. DOI: 10.1111/hsc.13853. 35599431 PMC10083988

[bibr22-14713012251342059] Gilleard C. Higgs P. (2020). Precarity and the assumption of rising insecurity in later life: A critique. Ageing and Society, 40(9), 1849–1866. DOI: 10.1017/s0144686x19000424.

[bibr23-14713012251342059] Grenier A. Hatzifilalithis S. Laliberte-Rudman D. Kobayashi K. Marier P. Phillipson C. (2020a). Precarity and aging: A scoping review. The Gerontologist, 60(8), e620–e632. DOI:10.1093/geront/gnz135 31675418

[bibr54-14713012251342059] Grenier A Phillipson C. (2023). Precarity and dementia. In Ward R. Sandberg L. (Eds.), Critical Dementia Studies (pp. 119–135). Routledge.

[bibr24-14713012251342059] Grenier A. Phillipson C. Settersten R. A. (2020b). Precarity and ageing: New perspectives for social gerontology. In Precarity and ageing (pp. 1–16). Policy Press.

[bibr25-14713012251342059] Hall K. (2023). Care precarity among older British migrants in Spain. Ageing and Society, 43(8), 1915–1933. DOI: 10.1017/S0144686X21001392.

[bibr27-14713012251342059] Hillman A. Jones I. R. Quinn C. Pentecost C. Stapley S. Charlwood C. Clare L. (2023). The precariousness of living with, and caring for people with, dementia: Insights from the IDEAL programme. Social Science & Medicine, 331, Article 116098. DOI: 10.1016/j.socscimed.2023.116098 37480697

[bibr28-14713012251342059] Javanparast S. Naqvi S. K. A. Mwanri L. (2020). Health service access and utilisation amongst culturally and linguistically diverse populations in regional South Australia: A qualitative study. Rural and Remote Health, 20(4), 1–15. DOI: 10.22605/RRH5694. 33207914

[bibr29-14713012251342059] Javed A. Lee C. Zakaria H. Buenaventura R. D. Cetkovich-Bakmas M. Duailibi K. Ng B. Ramy H. Saha G. Arifeen S. Elorza P. M. Ratnasingham P. Azeem M. W. (2021). Reducing the stigma of mental health disorders with a focus on low-and middle-income countries. Asian Journal of Psychiatry, 58, Article 102601. DOI: 10.1016/j.ajp.2021.102601 33611083

[bibr30-14713012251342059] Jeon Y.-H. Shin M. Smith A. Beattie E. Brodaty H. Frost D. Hobbs A. Kotting P. Petrie G. Rossor M. Thompson J. Vickers J. Waters D. (2021). Early implementation and evaluation of StepUp for Dementia research: An Australia-wide dementia research participation and public engagement platform. International Journal of Environmental Research and Public Health, 18(21), Article 11353. DOI: 10.3390/ijerph182111353. 34769871 PMC8583565

[bibr31-14713012251342059] Jones I. R. (2017). Social class, dementia and the fourth age. Ageing, Dementia and the Social Mind, 39(2), 128–141. DOI: 10.1111/1467-9566.12520. 28177145

[bibr32-14713012251342059] Khatri R. B. Assefa Y. (2022). Access to health services among culturally and linguistically diverse populations in the Australian universal health care system: Issues and challenges. BMC Public Health, 22(1), 880. DOI: 10.1186/s12889-022-13256-z. 35505307 PMC9063872

[bibr33-14713012251342059] Lin P. J. Daly A. T. Olchanski N. Cohen J. T. Neumann P. J. Faul J. D. Fillit H. M. Freund K. M. (2021). Dementia diagnosis disparities by race and ethnicity. Medical Care, 59(8), 679–686. DOI: 10.1097/MLR.0000000000001577. 34091580 PMC8263486

[bibr34-14713012251342059] Link B. G. Phelan J. C. (2001). Conceptualizing stigma. Annual Review of Sociology, 27(1), 363–385. DOI: 10.1146/annurev.soc.27.1.363.

[bibr35-14713012251342059] Lisko I. Kulmala J. Annetorp M. Ngandu T. Mangialasche F. Kivipelto M. (2021). How can dementia and disability be prevented in older adults: Where are we today and where are we going? Journal of Internal Medicine, 289(6), 807–830. DOI: 10.1111/joim.13227. 33314384 PMC8248434

[bibr36-14713012251342059] Low L. F. Barcenilla-Wong A. L. Brijnath B. (2019). Including ethnic and cultural diversity in dementia research. The Medical Journal of Australia, 211(8), 345–346. DOI: 10.5694/mja2.50353. 31559640

[bibr37-14713012251342059] Nadasen P. (2021). Rethinking care work: (Dis)Affection and the politics of caring. Feminist Formations, 33(1), 165–188. DOI: 10.1353/ff.2021.0008.

[bibr38-14713012251342059] NHMRC National Institute for Dementia Research . (2020). Culturally and linguistically diverse (CALD) Dementia research action plan. Available at: https://www.nhmrc.gov.au/sites/default/files/documents/attachments/Dementia/CALD-Action-Plan.pdf

[bibr55-14713012251342059] Nielsen T.R Nielsen D.S Waldemar G. (2021). Barriers in access to dementia care in minority ethnic groups in Denmark: a qualitative study. Aging & mental health, 25(8), 1424–1432. DOI:10.1080/13607863.2020.1787336 32619352

[bibr39-14713012251342059] Odzakovic E. Kullberg A. Hellström I. Clark A. Campbell S. Manji K. Rummery K. Keady J. Ward R. (2021). ‘It’s our pleasure, we count cars here’: An exploration of the ‘neighbourhood-based connections’ for people living alone with dementia. Ageing and Society, 41(3), 645–670.

[bibr40-14713012251342059] Pham T. T. L. Berecki-Gisolf J. Clapperton A. O’Brien K. S. Liu S. Gibson K. (2021). Definitions of culturally and linguistically diverse (CALD): A literature review of epidemiological research in Australia. International Journal of Environmental Research and Public Health, 18(2), 737. DOI: 10.3390/ijerph18020737. 33467144 PMC7830035

[bibr41-14713012251342059] Prince M. Ali G. C. Guerchet M. Prina A. M. Albanese E. Wu Y. T. (2016). Recent global trends in the prevalence and incidence of dementia, and survival with dementia. Alzheimer’s Research & Therapy, 8, 1–13. DOI:10.1186/s13195-016-0188-8 PMC4967299 27473681

[bibr42-14713012251342059] Rewerska-Juśko M. Rejdak K. (2020). Social stigma of people with dementia. Journal of Alzheimer's Disease, 78(4), 1339–1343. DOI: 10.3233/jad-201004. 33185610

[bibr43-14713012251342059] Sagbakken M. Spilker R. S. Ingebretsen R. (2018). Dementia and migration: Family care patterns merging with public care services. Qualitative Health Research, 28(1), 16–29. DOI: 10.1177/1049732317730818. 28918700

[bibr44-14713012251342059] Sakai E. Y. Carpenter B. D. (2011). Linguistic features of power dynamics in triadic dementia diagnostic conversations. Patient Education and Counseling, 85(2), 295–298. DOI: 10.1016/j.pec.2010.09.020. 21030193 PMC3097295

[bibr45-14713012251342059] Shanley C. Boughtwood D. Adams J. Santalucia Y. Kyriazopoulos H. Pond D. Rowland J. (2012). A qualitative study into the use of formal services for dementia by carers from culturally and linguistically diverse (CALD) communities. BMC Health Services Research, 12, 1–11. DOI:10.1186/1472-6963-12-354 23043332 PMC3523018

[bibr46-14713012251342059] Shatnawi E. Steiner-Lim G. Z. Karamacoska D. (2023). Cultural inclusivity and diversity in dementia friendly communities: An integrative review. Dementia, 22(8), 2024–2046. DOI: 10.1177/14713012231206292. 37871120 PMC10644696

[bibr47-14713012251342059] Siette J. Meka A. Antoniades J. (2023). Breaking the barriers: Overcoming dementia-related stigma in minority communities. Frontiers in Psychiatry, 14, Article 1278944. DOI: 10.3389/fpsyt.2023.1278944 PMC10765564 38179250

[bibr48-14713012251342059] Todorova I. Turner H. Castaneda-Sceppa C. Young D. Bonner A. (2016). “I do it with love” engagement in caring for people with dementia. Global Qualitative Nursing Research*, *3, Article 2333393616668634. DOI: 10.1177/2333393616668634 PMC5415280 28508019

[bibr49-14713012251342059] Tong A. Sainsbury P. Craig J. (2007). Consolidated criteria for reporting qualitative research (COREQ): A 32-item checklist for interviews and focus groups. International Journal for Quality in Health Care: Journal of the International Society for Quality in Health Care, 19(6), 349–357. DOI: 10.1093/intqhc/mzm042. 17872937

[bibr50-14713012251342059] Tuffour I. Ganga G. (2024). Dementia: A call for a paradigm shift in pre-registration nurse education. Cambridge Prisms. Global Mental Health, 11, Article e2. DOI: 10.1017/gmh.2023.80 PMC10808974 38283879

[bibr51-14713012251342059] Wang Q. Xiao X. Zhang J. Jiang D. Wilson A. Qian B. Song P. Yang Q. (2023). The experiences of East Asian dementia caregivers in filial culture: A systematic review and meta-analysis. Frontiers in Psychiatry, 14, Article 1173755. DOI: 10.3389/fpsyt.2023.1173755 PMC10160681 37151975

[bibr52-14713012251342059] Wilson T. Temple J. Brijnath B. Utomo A. McDonald P. (2022). The ageing of Asian migrant populations in Australia: Projections and implications for aged care services. Asian Population Studies, 18(1), 61–86. DOI: 10.1080/17441730.2021.1953689.

[bibr53-14713012251342059] World Health Organisation (WHO) (2021). Global status report on the public health response to dementia *.* Available at: https://digitalcommons.fiu.edu/cgi/viewcontent.cgi?article=1962&context=srhreports

