# Supplementary material for: Power, class, and gender in dementia care: Stories of carer precariousness from culturally and linguistically diverse female family carers in Australia
Source: Dementia (London). 2025 May 11;25(2):281–96. doi: 10.1177/14713012251342059 (PMC12816398; doi:10.1177/14713012251342059)
Supplement: Supplemental Material - Power, class, and gender in dementia care: Stories of carer precariousness from culturally and linguistically diverse female family carers in Australia [file sj-pdf-1-dem-10.1177_14713012251342059.pdf]

Supplementary Materials

S1. Consolidated criteria for reporting qualitative studies (COREQ): 32-item checklist.

**No**	**Item**	**Guide questions/description**	**Response/page**
**Domain 1: Reflexivity**			
Personal Characteristics			
1.	Interviewer	Which author/s conducted the interview?	PhD/MCl in Psy
2.	Credentials	What were the researcher's credentials? *E.g. PhD*	PhD<br>PhD/MCl in Psy<br>PhD<br>PhD
3.	Occupation	What was their occupation at the time of the study?	Research Fellow.
4.	Gender	Was the researcher male or female?	Female
5.	Experience and training	What experience or training did the researcher have?	p.9
Relationship with participants			
6.	Relationship established	Was a relationship established prior to study commencement?	No
7.	Participant knowledge of interviewer	What did the participants know about the researcher? e*.g. personal goals, reasons for doing the research*	Yes, p.9
8.	Interviewer characteristics	What characteristics were reported about the interviewer? e.g. *Bias, assumptions, reasons and interests in the research topic*	p.9
**Domain 2: study design**			
Theoretical framework			
9.	Methodological orientation and Theory	What methodological orientation was stated to underpin the study? *e.g. grounded theory, discourse analysis, ethnography, phenomenology, content analysis*	Yes, thematic p.7
Participant selection			
10.	Sampling	How were participants selected? *e.g. purposive, convenience, snowball*	Purposive, p.8
11.	Method of approach	How were participants approached? e*.g. face-to-face, telephone, mail, email*	Email & Phone, p.8
12.	Sample size	How many participants in the study?	Thirteen p.8
13.	Non-participation	How many people refused to participate or dropped out?	Nil,
Setting			
14.	Setting of data collection	Where was the data collected? e*.g. home, clinic, workplace*	Online, p.8
15.	Presence of others	Was anyone else present besides the participants and researchers?	No.

16.	Description of sample	What are the important characteristics of the sample? *e.g. demographic data, date*	p.7
Data collection			
17.	Interview guide	Were questions, prompts, guides provided by the authors? Was it pilot tested?	No pilot testing.
18.	Repeat interviews	Were repeat interviews carried out? If yes, how many?	No
19.	Audio/visual recording	Did the research use audio or visual recording to collect the data?	Yes, p.9
20.	Field notes	Were field notes made?	No
21.	Duration	What was the duration of the interviews?	60-90 mins, p.9
22.	Data saturation	Was data saturation discussed?	No
23.	Transcripts returned	Were transcripts returned to participants for comment and/or correction?	No,
**Domain 3: analysis & findings**			
Data analysis			
24.	No. of coders	How many data coders coded the data?	One, p.9
25.	Description of the coding tree	Description of the coding tree?	No
26.	Derivation of themes	Were themes identified in advance or derived from the data?	No
27.	Software	What software, if applicable, was used to manage the data?	Nil
28.	Participant checking	Did participants provide feedback on the findings?	No.
Reporting			
29.	Quotations presented	Were participant quotations presented to illustrate the themes / findings? Was each quotation identified? e.*g. participant ID*	Yes, Participant ID was included
30.	Data and findings consistent	Was there consistency between the data presented and the findings?	Yes, p.9-14
31.	Clarity of major themes	Were major themes clearly presented in the findings?	Yes.
32.	Minor themes	Were minor themes presented?	No
